# Implied Dynamics Biases the Visual Perception of Velocity

**DOI:** 10.1371/journal.pone.0093020

**Published:** 2014-03-25

**Authors:** Barbara La Scaleia, Myrka Zago, Alessandro Moscatelli, Francesco Lacquaniti, Paolo Viviani

**Affiliations:** 1 Laboratory of Neuromotor Physiology, IRCCS Santa Lucia Foundation, Rome, Italy; 2 Department of Cognitive Neuroscience, University of Bielefeld, Bielefeld, Germany; 3 Department of Systems Medicine, University of Rome Tor Vergata, Rome, Italy; 4 Centre of Space Bio-Medicine, University of Rome Tor Vergata, Rome, Italy; University Medical Center Goettingen, Germany

## Abstract

We expand the anecdotic report by Johansson that back-and-forth linear harmonic motions appear uniform. Six experiments explore the role of shape and spatial orientation of the trajectory of a point-light target in the perceptual judgment of uniform motion. In Experiment 1, the target oscillated back-and-forth along a circular arc around an invisible pivot. The imaginary segment from the pivot to the midpoint of the trajectory could be oriented vertically downward (consistent with an upright pendulum), horizontally leftward, or vertically upward (upside-down). In Experiments 2 to 5, the target moved uni-directionally. The effect of suppressing the alternation of movement directions was tested with curvilinear (Experiment 2 and 3) or rectilinear (Experiment 4 and 5) paths. Experiment 6 replicated the upright condition of Experiment 1, but participants were asked to hold the gaze on a fixation point. When some features of the trajectory evoked the motion of either a simple pendulum or a mass-spring system, observers identified as uniform the kinematic profiles close to harmonic motion. The bias towards harmonic motion was most consistent in the upright orientation of Experiment 1 and 6. The bias disappeared when the stimuli were incompatible with both pendulum and mass-spring models (Experiments 3 to 5). The results are compatible with the hypothesis that the perception of dynamic stimuli is biased by the laws of motion obeyed by natural events, so that only natural motions appear uniform.

## Introduction

Humans make striking perceptual mistakes in judging even the simplest kinematics of visual stimuli. Thus, a spot moving at constant velocity along a rectilinear path is perceived as moving fast upon entering the visual field and then decelerating to a constant velocity [1]–[3]. Runeson [3] showed conversely that the motion is perceived as uniform when the velocity is mildly increasing at the onset and then levels off. Gross misperceptions also involve continuous movements. Johansson [4] asked to describe qualitatively the motion of a target moving back-and-forth sinusoidally. Observers reported that, aside from a slight deceleration at trajectory endpoints, velocity looked constant.

Runeson [3] argued that the perception of dynamic stimuli is attuned to the statistical properties of the environment, being biased by the laws of motion of natural events. The velocity of a visual stimulus would be estimated with reference to an ecological motion with a compatible trajectory. Insofar as velocity changes are consistent with a natural dynamic event, they would not be taken into account, and velocity would be perceived as approximately constant.

The idea that the expected dynamics of natural stimuli biases visual motion perception has been extensively tested in the case of biological movements [5]. It has been suggested that motion perception is influenced by implicit expectations about the properties of human voluntary movements [6]. Viviani and Stucchi [7] asked observers to adjust the velocity of a dot following regular or random curvilinear trajectories until the motion was perceived as uniform. The selected profiles were actually highly non-uniform, mimicking closely the kinematics of drawing movements in which instantaneous velocity and curvature covary according to the 2/3 Power Law [8]. This connection between visual perception and the velocity-curvature covariance has been confirmed for 2D [9] and 3D trajectories (where torsion also plays a role, [10]). Moreover, it has been shown that the 2/3 Power Law can be derived from the assumption that the equi-affine velocity of movements is constant [11]–[12].

The motions of masses under force fields afford another rich array of natural events that can bias perception. Gibson [13], Johannson [14] and Shepard [15] argued that perceiving is guided by long-enduring constraints in the external world. The further inference can been made that perceptual judgments result from a Bayesian decision process in which visual evidence is weighted by a statistical prior model of the natural environment, a larger weight being associated with events that occur more frequently [16]–[18]. Runeson's hypothesis [3] provides a plausible explanation for Johansson's observation [4] mentioned above, because the kinematics of the stimuli was consistent with that of a very simple physical system, namely a mass attached to a spring.

A more stringent test of Runeson's hypothesis is possible by considering stimuli compatible with more than one ecological model. The present study focuses on the perception of a very common visual template, the oscillation of an object around a pivot. Many real-world events, including biological movements, are described approximately by such a template that arises whenever a mass is subjected to elastic and/or gravitational forces. In particular, the perceived motion of the pendulum has received considerable attention. When friction is negligible, the instantaneous velocity of a suspended mass under Earth gravity depends only on its initial position and on the length of the pendulum rod. Moreover, in the small-angle approximation, the oscillation is harmonic with a period that depends only on the length of the rod.

The earliest evidence that the motion of the simple pendulum is perceptually salient was provided by measuring the accuracy with which one can point to the bob [19]. Pointing errors were found to be smaller for a normal upright pendulum accelerating downwards than for an upside-down pendulum artificially accelerating upwards. More recently, the extent to which visual perception is sensitive to departures from the relation between period and rod length has been investigated extensively [20]–[23]. When a pendulum swings faster or slower than normal, the naturalness ratings decrease with the extent to which the legitimate length-period relation is violated, and are affected by asymmetries in the oscillations [22]. Observers can distinguish patch-light displays of either a freely swinging or a hand-moved pendulum, despite identical periods and amplitudes, but recognition is impaired with an upside-down pendulum [24].

Under specific circumstances, gravity and elastic forces induce similar harmonic oscillations. In general, however, the two forces are very different. Unlike elastic tension, gravity is ubiquitous, almost constant at Earth surface, and acts invariably in the downward direction. In fact, the expected constraints imposed by gravity on object motion can affect visual perception more generally than in the simple oscillatory case [25]–[27]. If the perception of oscillatory motions was biased by the expected behavior of ecological models, velocity judgments on oscillating targets should depend not only on visual cues, but also the a priori likelihood that either gravitational or elastic forces (or both) are at work. Specifically, when the implied position of the pivot is vertically above the midpoint of the target trajectory, both gravitational and elastic models are compatible with the visual cues because the corresponding kinematics are almost indistinguishable. Therefore, observers should be misled into perceiving the harmonic velocity profile as constant. By contrast, as the orientation diverges from the downward vertical, the elastic model remains plausible but the gravitational pendulum model becomes increasingly implausible. Thus, velocity changes should become more salient and perceptual judgments less robust. Moreover, velocity changes should no longer be neglected when the stimulus is modified so that neither the elastic nor the pendulum model can be assumed as plausible models.

We designed six experiments to test these predictions and to investigate the perceptual consequences of modifying some of the features that are normally associated with the motion of a gravitational pendulum or a mass-spring system. We presented a spot moving along curvilinear or linear trajectories with different velocity profiles including both harmonic and constant velocity motion, and asked the observers to choose the profile that appeared most uniform. By varying the law of motion, the shape of the trajectory, and its orientation relative to gravity, we explored a significant range of possible departures from the canonical motion of a simple pendulum. In Experiment 1, the target oscillated back-and-forth along a circular arc. The effects of target kinematics and of the orientation of the trajectory relative to gravity were manipulated independently. In Experiments 2 to 5, the target moved uni-directionally. The effect of suppressing the alternation of movement directions that characterizes the motion of a pendulum was tested with curvilinear (Experiment 2 and 3) or rectilinear (Experiment 4 and 5) paths. In Experiments 1 to 5, no instruction was given concerning eye movements and observers were free to eye-track the target. Experiment 6 tested whether perceptual judgments are affected by the absence of extra-retinal signals related to eye movements. We replicated one orientation condition of Experiment 1, but participants were asked to hold the gaze on a fixation point.

## Experiment 1

The anecdotal report by Johansson [4] that to-and-fro motions with sinusoidally varying velocity look uniform is compatible with Runeson's general hypothesis on the perception of natural events (“only natural motions look constant”, [3] page 11). However, Johansson's study involved only rectilinear horizontal trajectories, which can be construed as representing a pendulum motion only in the limiting case of infinitely long rods. Moreover, he tested just one sinusoidal profile. The aim of our first experiment was to investigate thoroughly how the velocity of a pendulum is perceived by extending on three counts Johansson's experimental condition [4].

First, we presented stimuli moving along curvilinear trajectories with constant radius, which are always compatible with the motion of a pendulum. Second, rather than asking a qualitative judgment on just one velocity profile, we asked observers to select the most uniform motion from a full range of alternative profiles, which also included the uniform one. The range of velocity modulations was wide enough to exclude that perceptual misjudgments of the kind reported by Johansson [4] simply reflect a high threshold for detecting velocity changes [28]–[33]. Moreover, differences between adjacent velocity profiles in the psychophysical staircases were finely controlled in order to afford reliable estimates of both accuracy and precision of the perceptual judgments. Third, to investigate the role of gravity, we contrasted perceptual judgments across three orientations, i.e., normal upright, horizontal, and upside-down.

If perception relies heavily on statistical prior models of physics, and if “only natural motions look constant”, the harmonic motion should be chosen as prototype of uniform motion for the three stimulus orientations, which are all consistent with the assumption that oscillations are driven by elastic forces. However, if a prior related to the direction of gravity also affected perception, one would expect that the harmonic motion should be chosen with greater accuracy, precision and inter-individual consistency in the canonical upright orientation of a pendulum than in the other two orientations incongruent with gravity.

Note that, although the stimuli could be construed also as a schematic description of a voluntary biological movement (such as the back and forth swing of a limb), they were point-to-point motions with a constant curvature throughout and a reversal of velocity direction at the endpoints of the trajectory. One cannot expect the responses to comply with the 2/3 Power Law, which does not apply to such movements.

### Methods

#### Participants

Twelve participants (9 females and 3 males; 22.8±2.9 years old, mean ± SD) volunteered for the experiment. They were all right-handed (as assessed by a short questionnaire based on the Edinburgh scale), had normal or corrected-to-normal vision, and no past history of psychiatric or neurological diseases. All participants in this and the following experiments gave written informed consent to procedures approved by the Institutional Review Board of Santa Lucia Foundation, in conformity with the Declaration of Helsinki on the use of human subjects in research, but were otherwise unaware of the purpose of the experiments.

#### Kinematics of the target

The motion (hereafter PM_1g_) of an ideal simple pendulum under Earth gravity (g = 9.81 m s^−2^) obeys the differential equation:
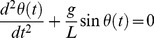
(1)where *θ(t)* is the angular displacement from the equilibrium position (*θ* = 0), and L is the rod length. With the initial conditions:
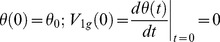
(2)the movement is an oscillation with period:
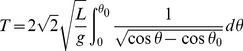
(3)Here we set L = 0.64823 m, θ_0_ = π/4. Thus, T = 1.68 s. In the following, A = 2θ_0_ = π/2 denotes the amplitude of the oscillation. Angular velocity (V_1g_ = dθ(t)/dt) was computed by solving Equation 1 numerically with a fourth-order Runge-Kutta algorithm. The maximum deviation between PM_1g_ and the law of motion computed in the small-angle approximation (harmonic function) was 0.7% and 1.3% for angular position and velocity, respectively. We use the term *Velocity* to indicate the unsigned time derivative of the angular displacement.

**Figure 1:**
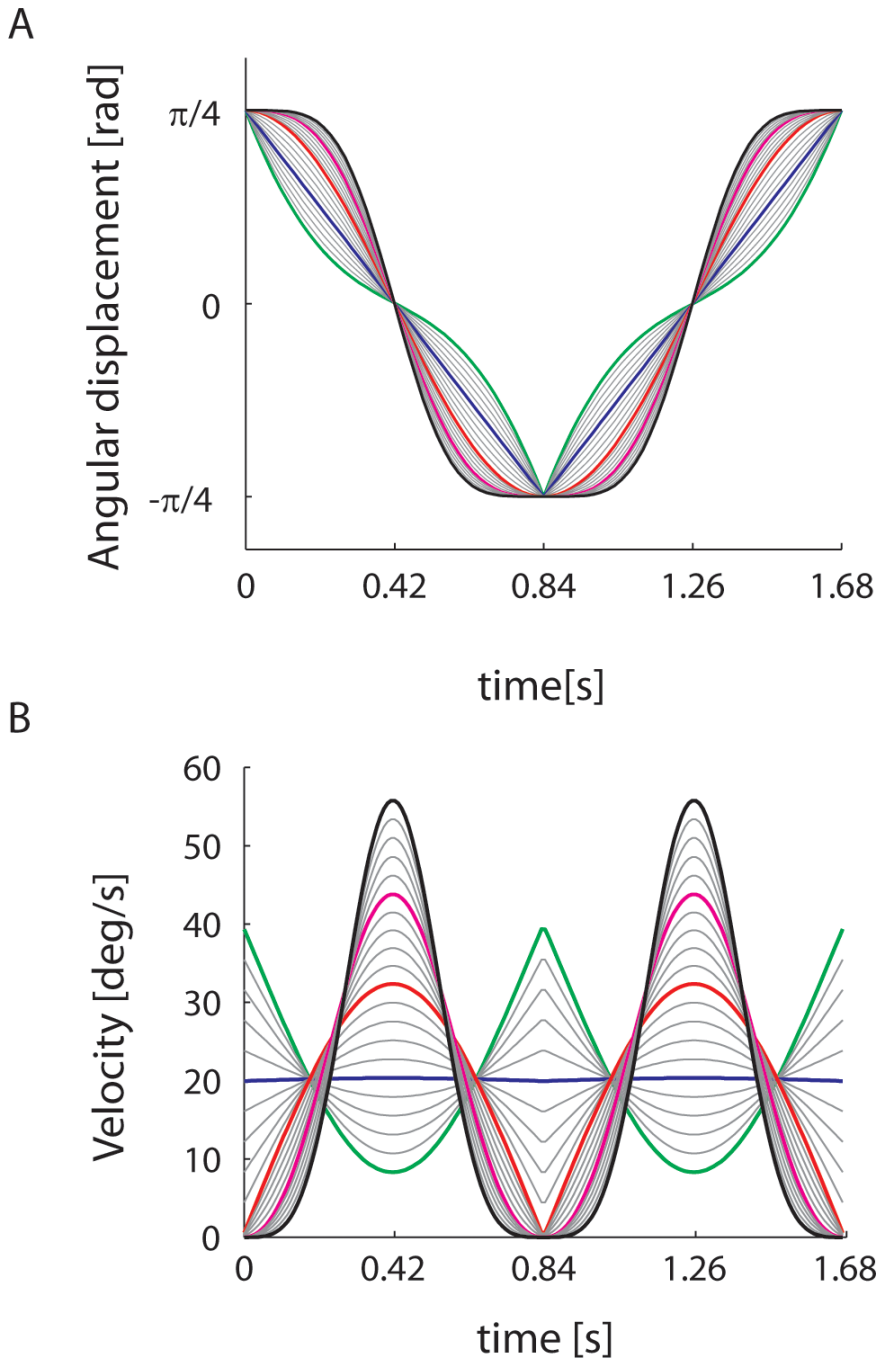
A: Angular displacement θ(t) of the target on the display for the 21 motion conditions. Thick traces denote the basic conditions (green: −1 g, blue: 0 g, red: 1 g, magenta: 2 g, black: 3 g). Thin traces denote the weighted combinations of the basic conditions. B: Visual angular velocity of the target for the 21 motion conditions. Same color codes as in A.

Using PM_1g_ as starting point, we generated 20 additional pendulum-like motions (PLM) with the same parameters (L, θ_0_ and T), but different velocity profiles (see Figure 1 and File S1, S2, S3). The procedure was as follows. First, we defined 4 basic motion conditions denoted as PLM_-1g_, PLM_0g_, PLM_2g_, and PLM_3g_. PLM_0g_ had a constant velocity V_0g_ throughout:
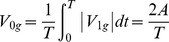
(4)


PLM_2g_ and PLM_3g_ had the following velocity profiles, respectively:
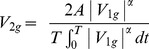
(5)




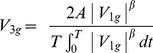
(6)The constants α and β were chosen so that V_2g_ (T/4)−V_1g_ (T/4) = V_1g_ (T/4)−V_0g_ (T/4), and V_3g_ (T/4)−V_2g_ (T/4) = V_2g_ (T/4)−V_1g_ (T/4), respectively. The labels 2 g and 3 g denote conditions with a maximum velocity twice and three times as large as 1 g, but do not describe a pendulum motion under a gravity level twice or three times as large as Earth's gravity. In fact the corresponding velocity profiles depart significantly from harmonic functions (see Figure 1B). Finally, PLM_−1g_ had the velocity profile V_−1g_ of a pendulum moving under reverse gravity:
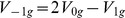
(7)In the second step of the procedure, the remaining 16 velocity profiles were interpolated as linear combinations of adjacent pairs of velocity profiles:
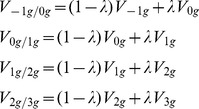
(8)with λ = 0.2, 0.4, 0.6, 0.8. In the following, the 21 velocity profiles will be referred to as [K_0_, K_1_,....K_20_], where K_0_ corresponds to −1 g, K_5_ to 0 g, K_10_ to 1 g, K_15_ to 2 g, K_20_ to 3 g, and the intermediate indexes denote the interpolated conditions. The time series of position coordinates of the pendulum endpoint was computed by numerical integration of the velocity. Round-off errors were minimized by computing 16800 position coordinates (10000 samples per second, over T = 1.68 s), and then under-sampling the sequence to match the vertical refresh rate of the display.

#### Apparatus and visual stimuli

Participants sat 0.58 m in front of a display (CRT EIZO Flexscan F980, active display size: 402 mm horizontal ×300 mm vertical, 38.3°×28° visual angle, 1600×1200 pixel resolution, 100 Hz vertical refresh rate) in a dimly illuminated room. The height of the chair was adjusted so that participant's eyes were ∼10° above the display midpoint. Responses were entered by pressing one of 4 keys (button-box Empirisoft Corp.) labeled (in Italian) “Start”, “Forward”, “Backward”, and “OK” (see *Task and procedures*). Stimuli were programmed in C++ and rendered using Autodesk Maya 2009 (Autodesk Inc) with a PNY NVIDIA Quadro FX5600 graphics card.

**Figure 2:**
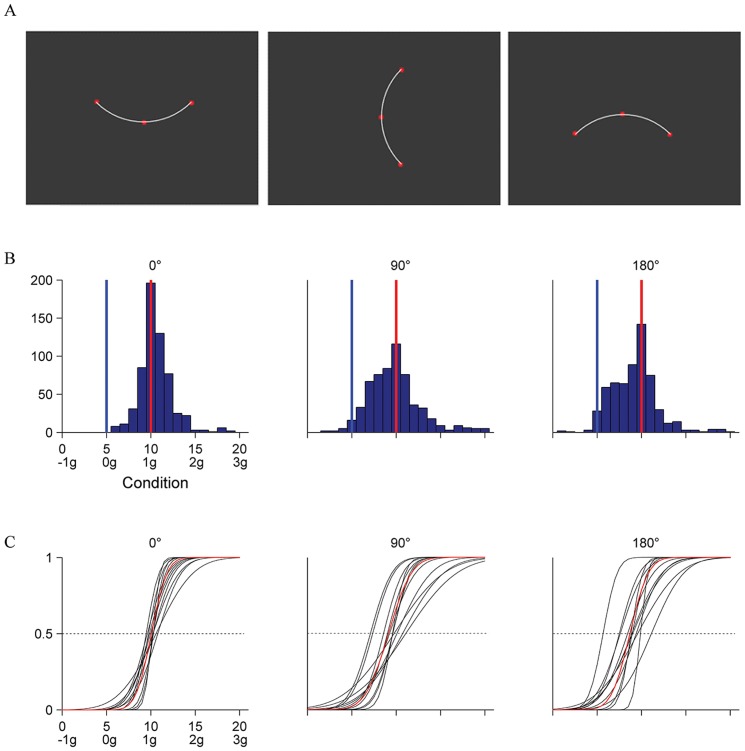
Experiment 1. A: Schematic of target trajectories. A red dot oscillated back and forth along a circle arc according to the kinematics of one of the 21 motion conditions of Figure 1 (trajectory is shown for illustrative purposes only). The orientation of the target path varied in different sessions, so that the virtual pendulum at equilibrium was upright (0°, left panel), horizontal (90°, middle panel), or upside-down (180°, right panel). Target and trajectories are not drawn to scale, but are plotted in the correct coordinates relative to the display. B: Distribution histograms of the responses (pooled over participants) for each orientation of the pendulum. Abscissae: motion conditions labeled both as K_i_ and as g-multiples. Ordinates: number of responses. Blue bars: ideal correct response; red bars: distribution medians. C: CDFs estimated by the GLMM for each participant (black) and for the population (red).

The target stimulus was a red dot (0.34°-diameter, chromaticity coordinates x = 0.538, y = 0.287 in CIE System, luminance 23.46 cd/m^2^), moving against a uniformly bluish-purple background (chromaticity x = 0.252, y = 0.177, luminance 3.85 cd/m^2^). Luminance and chromaticity were measured with a Tektronix J17 LumaColor photometer. In each trial, the target oscillated back-and-forth along the arc of a circle (10.74°-radius, 17.27 cm-length, 16.51°-secant) around an invisible pivot (Figure 2A). The imaginary segment from the pivot to the midpoint of the trajectory could be oriented vertically downward (0° orientation), horizontally leftward (90° orientation), or vertically upward (180° orientation). The kinematics of the target was identical for all orientations, but the direction of *g* in Eq. 1 was rotated consistent with the rotation of the trajectory. Thus, *g* accelerated the target downwards, leftwards, and upwards in the 0°, 90° and 180° orientations, respectively. With the kinematics of Eq. 1 and T = 1.68 s, the target moved as the bob of a virtual pendulum of length L, located at a distance of 3.5 m from the observer. Movies S1–S21 in File S1 show a down-sampled version of the stimuli presented in the 0° orientation. Movies S22–S42 in File S2 show the movies for the 180° orientation.

#### Detection of accelerating motions

Human sensitivity to visual accelerations is based on the comparison of velocity estimates averaged over short, successive time windows [30]–[33], and is known to be mediocre [32]–[33]. Thus, we verified whether accelerations in our stimuli were above the threshold reported in the literature. For all stimuli we computed the ratio |*V*_2_−*V*_1_|/*V*_average_, where *V*_1_ and *V*_2_ are the angular velocity at 0 and π/4, respectively, and *V*_average_ is the average velocity over that segment. The ratio ranged between about 30% (K_4_, K_6_) and 275% (K_20_). For all kinematic profiles except K_5_ (constant velocity), the ratio exceeded the detection threshold of 25% reported for stimuli with comparable duration and modulation frequency [30]–[31].

#### Protocol

Participants were tested in a counterbalanced order in three experimental sessions about 15 days apart. In each session, we presented one of the three orientations defined above (block design). In all trials, the target initial position was at π/4 clockwise relative to the trajectory midpoint. The target oscillated continuously with the same law of motion until the participant intervened either to modify the target kinematics (see below), or to end the trial with a response. The initial kinematics for a trial was selected pseudo-randomly from the set [K_2_, K_6_, K_10_, K_14_, K_18_,], with the constraint that successive trials could not have the same initial kinematics. There were 5 (initial kinematics) × 10 (repetitions)  = 50 trials in each session.

#### Task and procedures

Before the experiment, participants read the following instructions (in Italian). “Upon pressing the “Start” button, a red dot will start moving to-and-fro on the display with a velocity profile chosen by the computer, and you can watch this movement as long as you wish. Your task is to change the velocity profile until it looks uniform, that is, until the dot appears to move at constant velocity over the entire trajectory. At any time during the presentation, you can change the velocity profile in opposite directions by pressing either the “Forward” or the “Backward” button. Beware that the task may require several changes. To identify the most uniform motion, you may need to switch repeatedly between “Forward” and “Backward”. When you are satisfied that the velocity is constant, press the OK button. The stimulus will disappear marking the end of the trial. To begin a new trial, press the “Start” button. All trials are similar, except for the fact that the initial velocity profile is different. Whenever you wish to pause, simply refrain from pressing the “Start” button”. Next, the experimenter performed two trials to demonstrate the effects of pressing the different buttons, but did not provide any information about the correct response. The experiment started immediately after this familiarization phase.

The effect of pressing the buttons was to move one step backward (K_i_ → K_i−1_) or forward (K_i_ → K_i+1_) within the ordered sequence [K_0_, K_1_, … K_20_]. If K_i+1_ or K_i−1_ fell outside the preset range of conditions, the computer replaced it randomly by one of the three conditions [K_6_, K_10_, K_14_]. Participants were informed about neither the number of steps in the sequence nor the condition they were currently exploring. No feedback about response accuracy was provided. No instruction was given concerning eye movements so that participants were free to eye-track the target. Responses were stored together with the preceding sequence of changes. On average, reaching a final decision required 12 adjustments. The typical duration of a session was 40 min. None of the participants reported any sensation of motion-in-depth.

#### Data analysis and modeling

In the following, we use a vector notation (boldface) to denote the performance for the three orientations of the trajectory. Let the 3-vectors **P** and **W** indicate the probability P_nij_ that participant i judged as uniform the kinematics K_j_ with orientation n, and the associated cumulative distribution function (CDF) W_nij_, respectively. Moreover, in order to treat the stimuli as an ordinal random variable, we define the 3-vector **I** = [0, 1, … 20; 0, 1, … 20; 0, 1, …20] indexing the corresponding kinematics. The results were analyzed at both population and individual level. At population level (responses pooled over all participants, starting conditions and repetitions), the effect of orientation was estimated in several different ways. First, by computing the median (**M**) and the interquartile range (**IQR**) of the index of the kinematics judged as uniform. Second, global estimates of the central tendency index and of the variability were derived from a Probit analysis of **W**: 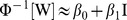
(9)where Ф^−1^ is the probit link function, **β_0_** the intercept and **β_1_** the slope of the linear regression. Goodness of fit was assessed by testing that the deviance was not significantly different from 0 [34]. At individual level, we repeated the probit analysis of the CDF separately for each participant.

As a further analysis at population level, we applied to the data the Generalized Linear Mixed Model (GLMM, [35]–[36]). The GLMM extends the probit analysis (Eq. 9) to clustered categorical data by framing the overall variability into fixed-effects predictors (the vector **I** of experimental kinematics) and random-effects predictors (to account for the variability among participants). For participant j the complete model, which also includes the interactions between fixed and random factors, is:
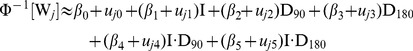
(10)where **W**_j_ is the CDF of participant j, **D_90_** and **D_180_** are dummy variables coding for the orientation (with respect to the 0° orientation reference), **I·D_90_** and I·**D_180_** are the interaction terms, *β_0_*, …, *β_5_* are the fixed-effects coefficients (independent of participants), and *u_j0_*, …, *u_j5_* are the random-effects coefficients for participant j. In particular, *β_1_* estimates the precision of the response in the reference (0°) orientation (the higher is β_1_, the greater the precision), *β_2_* (*β_3_*) tests whether the intercept is significantly different between the reference and the 90° (180°) orientation, and *β_4_* (*β_5_*) tests the same difference for the slope. The model was applied twice, by taking as reference first the 0° orientation (as detailed above), and then the 90° orientation (with the appropriate dummy variables **D**). The model was fitted to the CDF's using the R package ‘lme4’ [37]. The significance of the coefficients (two-sided P-values) was assessed by means of the Wald statistics:
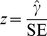
(11)where 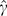 and SE are the parameter estimate and its standard error, respectively.

Median and JND for the population were again estimated from GLMM using the R package “MERpsychophysics” ([36], www.mixedpsychophysics.wordpress.com).

The medians of the CDFs were estimated as:
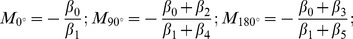
(12)The JNDs were estimated as the difference between the median and the motion condition for which the fitted model predicts a.75 response probability:
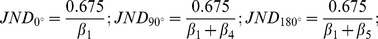
(13)95% confidence intervals of the parameters were computed by means of the delta method [38]. Statistical significance for all tests was set at α = 0.05.

#### Data availability

For this and the following experiments, the authors make freely available any materials and information described in this paper that may be reasonably requested by others for the purpose of academic, non-commercial research. Please contact b.lascaleia@hsantalucia.it, lacquaniti@med.uniroma2.it or m.zago@hsantalucia.it.

### Results and Discussion

At the population level, there was no significant effect of repetition on the response median (Kruskal-Wallis, 0° orientation: P = .07; 90° orientation: P = .5719; 180° orientation: P = .1698). There was a significant (P<.001) attractive effect of the initial kinematics on the median. However, the size of the effect was very small, the difference between maximum and minimum median values, computed separately for each starting condition, being equal to 1. Therefore, in the subsequent analyses the results were pooled across repetitions and starting conditions.

Figure 2B shows for each orientation of the trajectory (see Figure 2A) the distribution histograms of the response variable (N = 600, 12 [participant] ×5 [starting condition] ×10 [repetition]). For all three orientations, responses tended to cluster around the kinematic profile K_10_ which simulated the effects of a virtual gravity (1 g) acting downwards (0° orientation), leftwards (90° orientation), or upwards (180° orientation). However, the specific distribution of the responses differed as a function of orientation. In particular, the variability was smaller for the 0° orientation (IQR = 1) than for both the 90° (IQR = 3) and the 180° orientation (IQR = 3). Moreover, the proportion of trials in which velocity profiles close to K_5_ (constant absolute velocity) were judged as uniform was lower for the 0° orientation than for the other two orientations.

These results were confirmed by a probit analysis of the population CDFs of the response variable. There was a highly significant difference (Kolmogorov-Smirnov, P<.0001) between the CDF for orientation 0° and those for either 90° or 180° orientation. Both the median and the slope (response precision) were higher for 0° orientation than for the other two orientations. Differences remained significant (Kolmogorov-Smirnov, P<.05) even when the CDFs were computed after equalizing the individual medians. In contrast, the CDFs for 90° and 180° orientation appeared to have the same shape (Kolmogorov-Smirnov, P = .5212), the same variance (Ansari-Bradley, P = .086), but different median value (Kruskal-Wallis, P = .0013).

**Figure 3:**
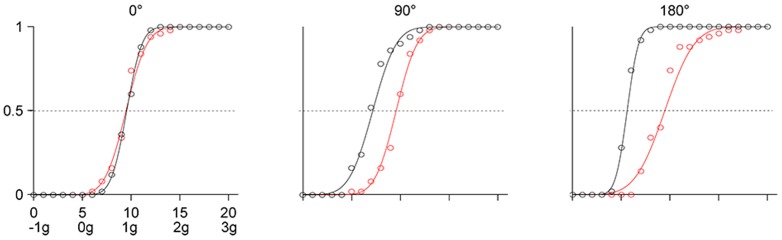
Experiment 1. CDFs of the responses in two participants (blue: S11, red: S12). Responses were pooled over all starting conditions and repetitions, and fitted with the probit function.

The effects of orientation were investigated further by modeling the individual CDFs of the response variable. Figure 2C shows the estimated CDFs for each participant (black) and for the population (red) using the GLMM (see *Data analysis and modeling*). With the only exception of *β_5_*, all parameters of the model were significantly different from zero (Wald Statistics, P<.05). Clearly, the responses were much more consistent among participants for 0° orientation than for the other two orientations. The difference across orientations is dramatized in Figure 3 by comparing the performance of two participants. Table 1 and 2 report for each orientation the estimated median and JND of the population CDFs. The median for the 0° orientation was not significantly different from K_10_ (1 g), but was significantly higher than the median for the other two orientations (90° and 180°). The medians for all stimulus orientations were significantly closer to K_10_ than to K_5_ (constant velocity). The slope of the CDF for orientation 0° was significantly higher than the slope for 90° orientation (*β_4_*, P<.005), whereas the slope was not significantly different between 0° and 180° orientations (*β_5_*, reference 0° orientation, P = .104) and between 90° and 180° orientations (*β_5_*, reference 90° orientation, P = .199).

**Table 1:** Experiment 1: Median values of the population CDFs estimated by GLMM for 0°, 90° and 180° orientation.

	Estimate	SE	Inferior CI	Superior CI
**0°**	10.0769696	0.1223967	9.8370765	10.316863
**90°**	9.154780	0.3085155	8.550101	9.759460
**180°**	8.659704	0.4762824	7.7262077	9.593200

SE: standard error. CI: 95% confidence intervals.

**Table 2:** Experiment 1: JND values of the population CDFs for 0°, 90° and 180° orientation.

	Estimate	SE	Inferior CI	Superior CI
**0°**	0.9391301	0.1276637	0.6889138	1.189346
**90°**	1.383318	0.1680376	1.053970	1.712665
**180°**	1.086853	0.1886720	0.7170624	1.456643

SE: Standard error; CI: 95% Confidence interval.

In the range [K_0_–K_9_], which includes the uniform motion case (K_5_), the kinematic profiles presented a discontinuity, because velocity does not go to zero at the endpoints of the trajectory (Figure 1B). The discontinuity may have been perceptually salient, and may have induced participants to reject these profiles as good prototypes of uniform motion. Indeed, almost never did participants select profiles in the range [K_0_–K_5_] (see Figure 2B). However, the hypothesis of a possible effect of discontinuities on the perceptual judgment is inconsistent with the strong tendency to select K_10_ rather than motions in the range [K_11_–K_20_] where no discontinuity existed. The small JND (<1) for the 0° orientation (Table 2) indicates that the observers were able to discriminate well the kinematic profiles of the conditions around K_10_. The difference in maximum angular velocity between K_10_ and either K_11_ or K_9_ was 2.1° s^−1^ (ΔV, see Methods), and the maximum angular velocity of K_10_ was 32.3° s^−1^ (see Figure 1B). Thus, the ability to discriminate between the maximum velocities of these conditions amounts to a Weber fraction of 6.5%, in keeping with previous estimates [39]–[40].

In sum, we found that a large misperception of target kinematics exists for all three orientations of the trajectory. Observers perceived as uniform a quasi-harmonic velocity profile that was strongly non-uniform, velocity changes being greater than 150% within each oscillation. The perceptual bias was significantly modulated by trajectory orientation. Responses for the 0° orientation clustered closer to the 1g-condition (K_10_) and were less variable across trials and participants than for the other two orientations (90° and 180°). In other words, the bias was stronger for a trajectory orientation consistent with the interpretation of the stimulus as a canonical upright pendulum accelerated by physical gravity. In the general discussion we elaborate the implications of these findings vis à vis the general notion that perception is influenced by the interpretation of the stimuli in terms of physical events. Two issues must be addressed. 1) Both gravitational and elastic oscillations are characterized by the inversion of movement direction at both ends of the trajectory and by the curvilinear trajectory of the target. The role of these features on velocity perception is investigated in the next 4 experiments. 2) The possible role of eye movements. The last experiment tests whether eliminating the possibility to track the target with eye movements affects velocity judgments.

## Experiment 2

Experiment 1 suggested that quasi-harmonic motions are perceived as uniform because back-and-forth oscillations provide a baseline template for velocity perception. One open question is whether a quasi-harmonic velocity modulation following a curvilinear trajectory continues to be perceived as constant even when inversions of movement direction are no longer present. This experiment addressed the question by presenting a target that followed the same path with the same velocity profiles as in the 0° orientation of Experiment 1, but the motion was unidirectional.

### Methods

#### Participants

Twelve participants (11 females, 1 male; age: 23.5±3.2 years) volunteered for the experiment. None of them had served in Experiment 1.

#### Apparatus, stimuli, and tasks

Apparatus, task, and general experimental procedures were the same as in Experiment 1. The target moved along the same trajectory and with the same 21 velocity profiles as in the 0° orientation of that experiment. However, motion was unidirectional, from left to right. Its duration was the same (840 ms) as that of a single sweep of the pendulum-like motion in Experiment 1. Upon reaching the rightmost position along the path, the target disappeared for 140 ms; then it reappeared in the leftmost position and immediately started a new sweep. The inter-sweep interval was chosen to permit the eyes to re-foveate the target with a saccade (typical duration for a horizontal 20°-saccade is about 70 ms, [41]). Instead, this interval was too short to afford the perception of a partially occluded back-and-forth motion, that is, an oscillation in which the return trajectory is masked. Before the experiment, the participant read instructions similar to those provided in Experiment 1, except that target motion was described as unidirectional. As in Experiment 1, no instruction was given concerning eye movements. Data analysis was the same as in the previous experiment, except that there was only one orientation, instead of 3 as in Experiment 1.

### Results and Discussion

**Figure 4:**
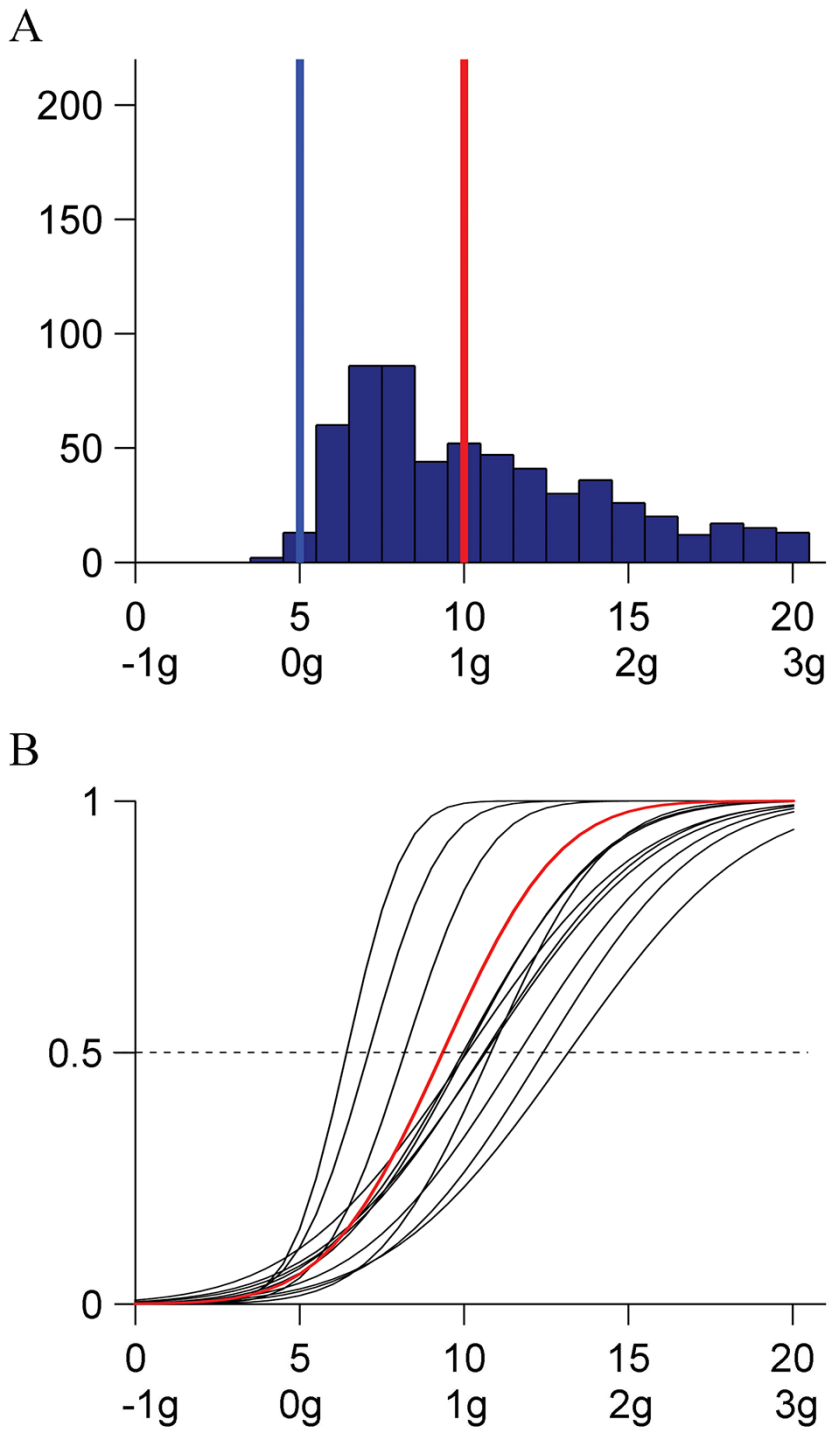
Experiment 2 (0° orientation, unidirectional). A: Distribution histogram of the responses (pooled over participants). B: CDFs for each participant and for the population. Same format as in Figure 2B.

There was no significant effect of repetition on the response median (Kruskal-Wallis, P = .7369), and a significant (P<.0001) effect of the starting condition in the sequence (the difference between the maximum and minimum median values was 4). Figure 4A shows the distribution histogram of all responses (N = 600, 12 [participant] ×5 [starting condition] ×10 [repetition]). Responses were scattered over a wider range of kinematic profiles (IQR = 6) than in Experiment 1. Figure 4B shows the CDFs estimated by the GLMM for each participant (black) and for the population (red). All parameters of the fit were significantly different from 0 (P<.0001). The median and JND of the population are reported in Table 3. The median was not significantly different from the profile K_10_ and was significantly higher than K_5_ (constant velocity). However, the JND was considerably higher than in the previous experiment. Taken together, the results of Experiment 1 and 2 show that a target moving with quasi-harmonic kinematics is perceived as uniform irrespective of whether full back and forth oscillation cycles or just repeated unidirectional half cycles are displayed. However, the responses tend to be considerably more variable in the latter than in the former case.

**Table 3:** Median and JND values of the population CDFs for Experiment 2.

Parameter	Estimate	SE	Inferior CI	Superior CI
**Median**	9.346431	0.7065479	7.961622	10.731239
**JND**	1.876218	0.2415015	1.402884	2.349553

## Experiment 3

In this experiment we asked whether the perceptual bias toward a quasi-harmonic velocity profile disappears when the trajectory is incompatible with any simple physical model capable of sustaining an oscillatory behavior. The velocity of the targets was modulated as in the previous experiments, but with a different trajectory. Here, the target moved along a circle without ever changing direction and was visible only in the bottom and top quadrants. These two segments of trajectory shown sequentially in time can be perceived as a partially occluded circular motion. Crucially, no physical system is compatible with a harmonic motion along a circular trajectory.

### Methods

#### Participants

Eleven participants (8 females and 3 males; age: 23.9±3.3 years) volunteered for the experiment. None of them had served in the previous experiments.

#### Apparatus, stimuli, and tasks

**Figure 5:**
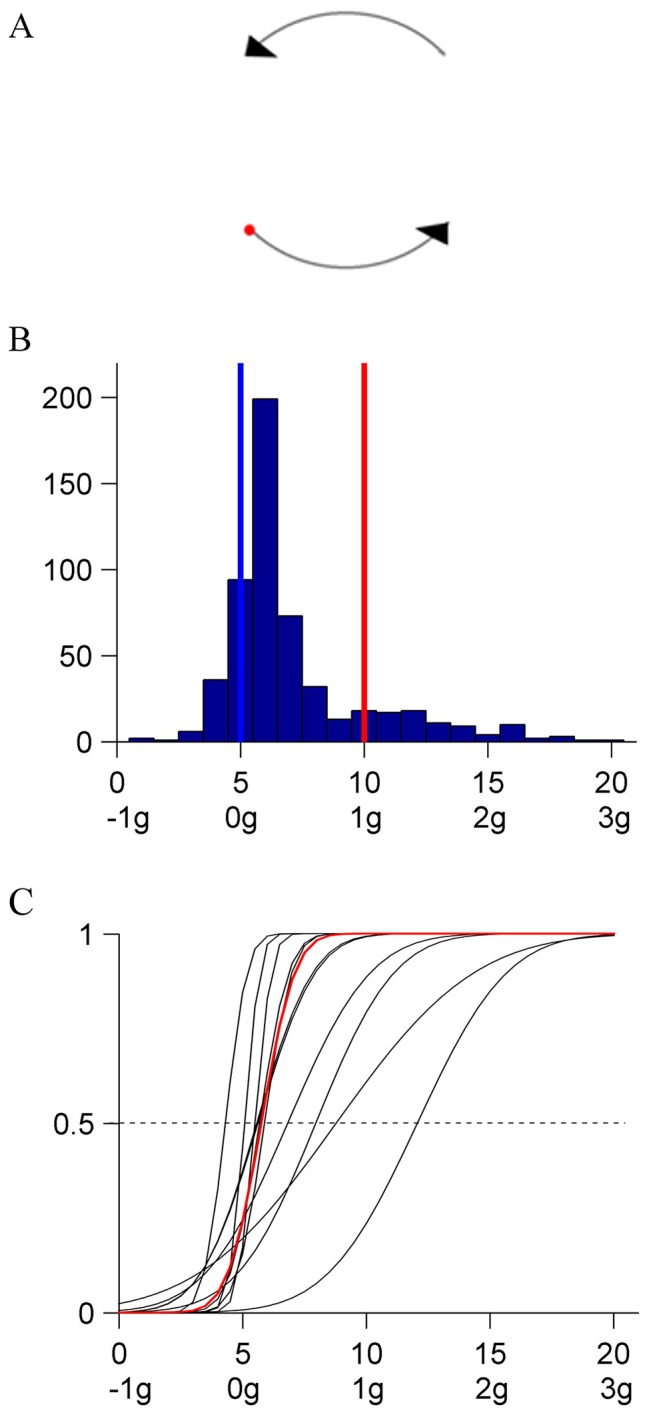
Experiment 3 (circular motion). A: Schematic of target trajectory. The target moved uni-directionally, anticlockwise on a circular trajectory. The target was visible only in the bottom and top quadrants. B: Distribution histogram of the responses (pooled over participants). C: CDFs for each participant and for the population. Same format as in Figure 2B.

Apparatus, task, and general experimental procedures were the same as in Experiment 1. The target moved uni-directionally, anticlockwise, along a circular trajectory centered on the display midpoint (Figure 5A). The radius of the trajectory was equal to the length of the virtual rod of Experiment 1. The period of rotation along the circle was 3.36 s, that is twice the period of a complete oscillation in Experiment 1. The target was visible only during half of this time, when it moved along the bottom and top circular segments (π/2-amplitude covered in 840 ms), which coincided with the target paths in the 0° and 180° orientations of Experiment 1, respectively. At the end of the visible segment, and before reappearing in the opposite segment, the target disappeared for 840 ms. Unlike in Experiment 2, the time interval during which the target remained invisible was compatible with a partial occlusion of a continuous motion. No instruction was given concerning eye movements.

### Results and Discussion

There was no significant effect of repetition on the median of the responses (Kruskal-Wallis, P = .7567), and a small but significant effect of the initial kinematics (difference between maximum and minimum median values  = 1, P<.001). Figure 5B shows the distribution histogram of all responses (N = 550, 11 [participant] ×5 [starting condition] ×10 [repetition]). The responses clustered around the median value corresponding to a very mildly changing velocity profile K_6_ (IQR = 3) (see Figure 1B). Participants were often able to correctly identify K_5_ as the constant velocity profile. Moreover, in sharp contrast with the previous experiments, only rarely did they choose the quasi-harmonic profile K_10_. Figure 5C shows the CDFs estimated by GLMM for each participant (black) and for the population (red). The parameters of the fit were significantly different from 0 (P<.0001). The median and the JND of the population CDF are reported in Table 4. The median was not significantly different from K_6_, while it was significantly lower than the medians in both previous experiments.

**Table 4:** Median and JND values of the population CDFs for Experiment 3.

Parameter	Estimate	SE	Inferior CI	Superior CI
**Median**	5.7560678	0.3612535	5.0480239	6.464112
**JND**	0.7183043	0.1543511	0.4157817	1.020827

In summary, the change in target configuration abolished almost completely the perceptual bias in favor of the harmonically modulated velocity profiles. Although the trajectory was partly occluded, the stimulus may have evoked the motion of a rotating system. As argued above, no simple such system exhibits harmonic oscillations. By contrast, a mass rotating under the effect of a central force - arguably the simplest physical model for such motion - does indeed move at constant velocity. Therefore, the results are still compatible with the general hypothesis that perception is permeable to pre-conceptions about the possible physical interpretation of the stimuli.

## Experiment 4

This and the next experiment investigate the role of trajectory curvature. In Experiment 2 we showed that even repeated unidirectional sweeps along a circular arc were sufficient to make participants perceive the quasi-harmonic motion of the target as uniform. Here we tested whether the same bias is present also when the target follows a horizontal rectilinear path.

### Methods

#### Participants

Twelve participants (8 females, 4 males; age: 23.8±3.4 years) volunteered for the experiment. Ten of them had participated also in Experiment 3. There were about 20 days between Experiment 3 and 4.

#### Procedures

The target moved on a linear, horizontal path from left to right, encompassing a visual angle of 17.85°. The path length was the same as that of the circular segments in Experiments 1 to 3. The midpoint coincided with the center of the display. Motion duration was the same (840 ms) as that of a single sweep of the pendulum-like motion in Experiment 1. As in Experiment 2, upon reaching the rightmost position along the path, the target disappeared for 140 ms and reappeared in the leftmost position to start a new sweep. Target velocity at any time along the linear trajectory was equal to the tangential velocity at the same time along the curvilinear path of Experiment 1. No instruction was given concerning eye movements. Movies S43–S63 in File S3 show a down-sampled version of the stimuli.

### Results and Discussion

**Figure 6:**
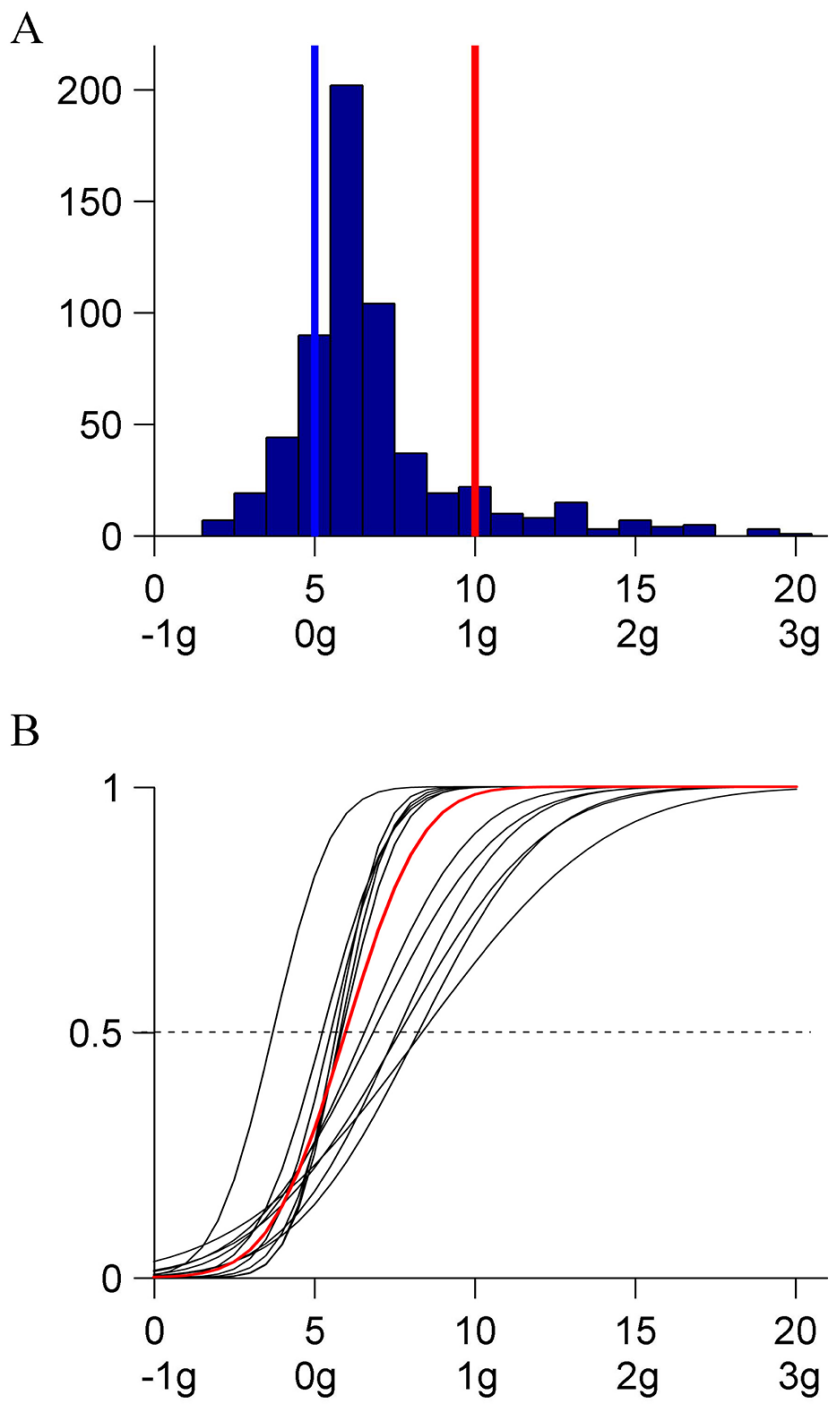
Experiment 4 (linear horizontal unidirectional). A: Distribution histogram of the responses (pooled over participants). B: CDFs for each participant and for the population. Same format as in Figure 2B.

There was no significant effect of repetition on the median of the responses (Kruskal-Wallis, P = .1507), and a small but significant (P<.005) effect of the initial kinematics (difference between maximum and minimum median  = 1). Figure 6A shows the distribution histogram of all responses (N = 600, 12 [participant] ×5 [initial kinematics] ×10 [repetition]). As in the previous experiment, the responses clustered around the median K_6_ (IQR = 2). Figure 6B shows the CDFs estimated by GLMM for each participant (black) and for the population (red). The parameters of the fit were statistically significant (P<.0001). The median and JND of the population CDF are reported in Table 5. The median was not statistically different from either K_6_ or the median of the previous experiment. Finally, the response in this and in the previous experiment had indistinguishable distributions (Kolmogorov-Smirnov, P = .6013). Therefore, the single difference with respect to Experiment 2 (rectilinear versus curvilinear trajectory) was responsible for eliminating almost completely the perceptual bias toward quasi-harmonic motion. As in the case of Experiment 3, this suggests that the bias disappeared because the stimuli no longer evoked an oscillating physical system.

**Table 5:** Median and JND values of the population CDFs for Experiment 4.

Parameter	Estimate	SE	Inferior CI	Superior CI
**Median**	5.969564	0.3365033	5.3100294	6.629098
**JND**	1.259944	0.1612077	0.9439827	1.575905

## Experiment 5

We tested whether the results of the previous experiment are confirmed when the rectilinear unidirectional motion is vertical instead of horizontal.

### Methods

#### Participants

Twelve participants (7 females, 5 males; age: 22.9±2.7 years) volunteered for the experiment. Five of them had participated also in Experiment 1. One of them had participated one year before also in Experiments 2 and 3.

#### Procedures

The target moved downward along a linear, vertical path of the same length as in Experiment 4 (17.85°). The midpoint coincided with the center of the display. Motion duration was the same (840 ms) as that of a single sweep of the pendulum-like motion in Experiment 1. Upon reaching the bottom position, the target disappeared for 140 ms and reappeared in the top position to start a new sweep. Target kinematics was the same as in Experiment 4. No instruction was given concerning eye movements.

### Results and Discussion

**Figure 7:**
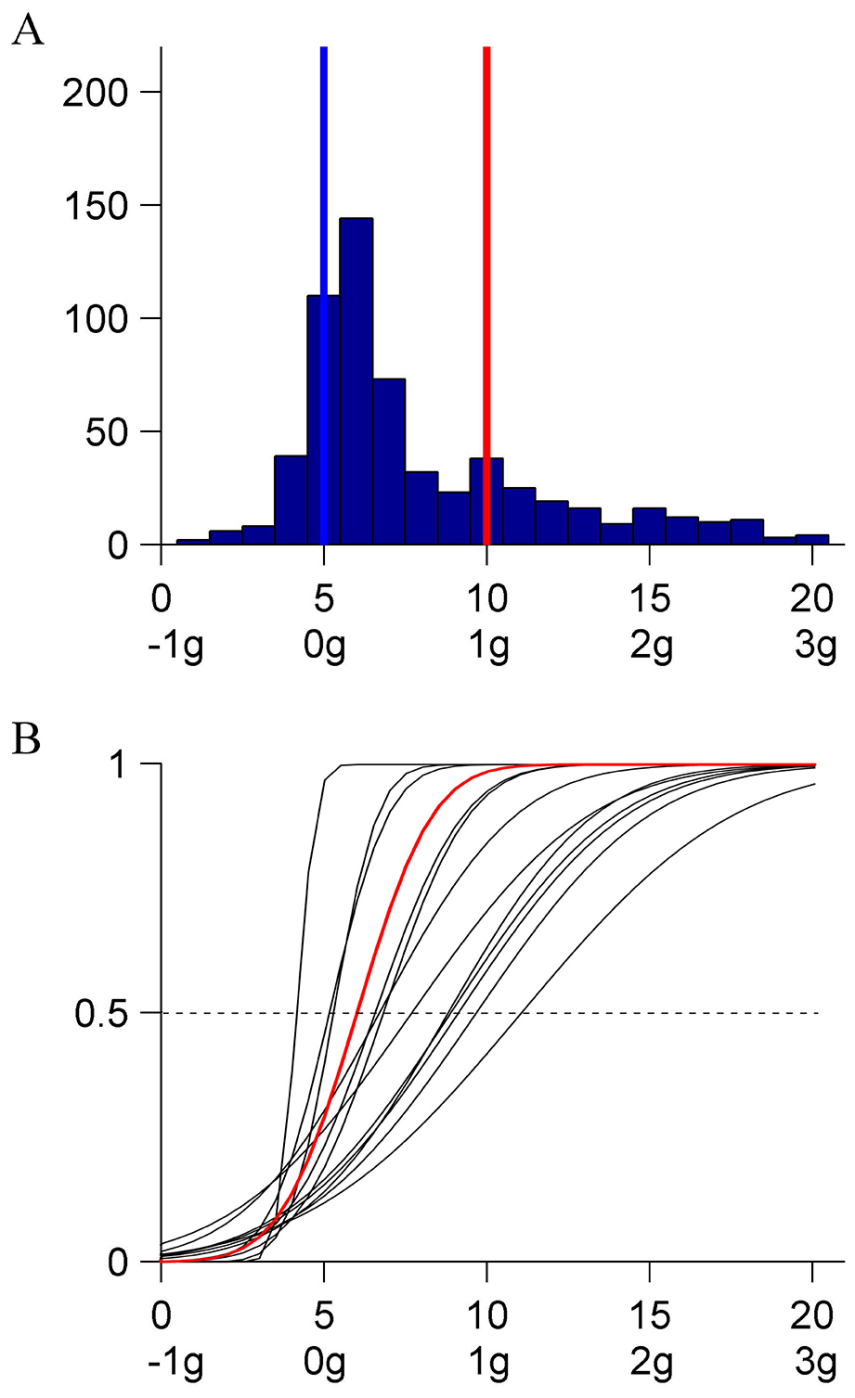
Experiment 5 (linear vertical unidirectional). A: Distribution histogram of the responses (pooled over participants). B: CDFs for each participant and for the population. Same format as in Figure 2B.

There was no significant effect of repetition on the median of the responses (Kruskal-Wallis, P = .4841), and a small but significant (P<.005) effect of the initial kinematics (difference between maximum and minimum median values  = 2). Observers were often able to correctly identify K_5_ as the constant velocity profile. Figure 7A shows the distribution histogram of all responses (N = 600, 12 [participant] ×5 [initial kinematics] ×10 [repetition]). As in Experiment 4, responses tended to cluster around the median K_6_ (IQR = 5). Figure 7B shows the CDFs estimated by GLMM for each participant (black) and for the population (red). The parameters of the fit were statistically significant (P<.0001). The median and JND of the population CDF are reported in Table 6. The median was not statistically different from K_6_. Taken together, the results of the last 3 experiments show that the perceptual bias towards quasi-harmonic motion is not inevitable, but requires that some cues in the stimuli evoke a context of an oscillatory physical system.

**Table 6:** Median and JND values of the population CDFs for Experiment 5.

Parameter	Estimate	SE	Inferior CI	Superior CI
**Median**	6.089861	0.8708472	4.3830320	7.796690
**JND**	1.273456	0.4235606	0.4432926	2.103620

## Experiment 6

In all previous experiments, no instructions were given concerning eye movements to participants, who were free to track the target. It has been reported that motions that can be construed as natural events (whether biological or inanimate) are easier to track with eye movements than motions deviating from such natural models [42]–[43]. Therefore, the ability to track the quasi-harmonic velocity profile better than the other profiles may have influenced the performance in Experiment 1. To address this issue, we replicated the 0° orientation condition of Experiment 1, by asking participants to hold the gaze on a fixation point.

### Methods

#### Participants

Six participants (5 females, 1 male; age: 23.6±3.9 years) volunteered for the experiment. Two of them had participated also in Experiment 1 about 30 days before.

#### Apparatus, stimuli, and tasks

The apparatus and general experimental procedures were the same as in Experiment 1. The target oscillated back and forth along the same trajectory and with the same 21 velocity profiles as in the 0° orientation condition. However, throughout each trial, participants were asked to fixate a white cross-hair (0.37°) located at the pivot of rotation of the target, 10° above the display midpoint. The head of the participants was stabilized with the help of a forehead-chin rest. The height of the chair and of the forehead-chin rest was adjusted individually so that the eyes were level with the fixation point. It is known that, in the range of values tested here (average angular velocity: 19.96° s^−1^), the retinal eccentricity of the target (10.74°) relative to the fovea does not alter velocity estimates substantially [44]–[45]. Moreover, the threshold detection for velocity changes is increased only slightly at comparable eccentricities [30], [46]. To verify that fixation was maintained steadily, observers were asked to perform also a secondary task. In 5 trials selected at random (mean inter-trial interval: 6 min), the cross-hair was briefly (500 ms) replaced by a small (0.25°) white letter. The sequence of 5 letters formed a word that participants were asked to report at the end of the experiment. The familiarization phase included a demonstration of the secondary task. To alleviate the fatigue of keeping a steady fixation, in addition to the pauses taken spontaneously by the participants, three 5-min pauses were forced after trial 20, 30 and 40.

### Results and Discussion

**Figure 8:**
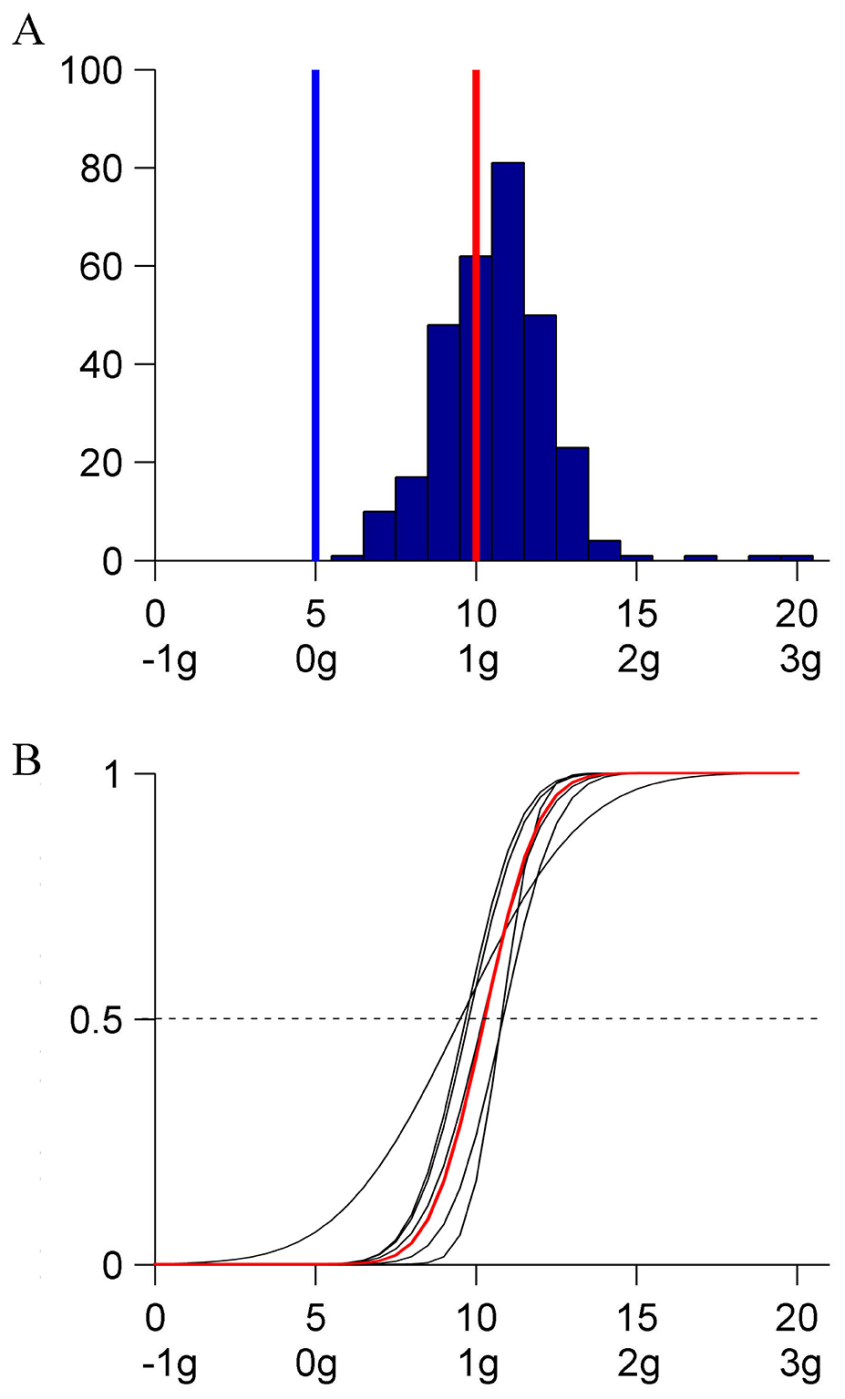
Experiment 6 (0° orientation, with eye fixation). A: Distribution histogram of the responses (pooled over participants). B: CDFs for each participant and for the population. Same format as in Figure 2B.

All participants correctly reported the words presented at the fixation point. Although the target moved in the periphery of the visual field, performance in the main task was very similar to that in Experiment 1. The median response depended on neither the initial kinematics (Kruskal-Wallis, P = .1146, responses pooled over all participants and repetitions), nor repetition (Kruskal-Wallis, P = .418). Figure 8A shows the histogram of all responses (N = 300, 6 [participant] ×5 [starting condition] ×10 [repetition]). As in the 0° orientation condition of Experiment 1, responses clustered around the kinematics K_10_ (IQR = 3). Figure 8B shows the CDFs estimated by GLMM for each participant (black) and for the population (red). The parameters of the fit were statistically significant (P<.0001). The median and JND of the population are reported in Table 7. The median was not significantly different either from K_10_ or from the corresponding median in the 0°orientation condition of Experiment 1. The probit fit to the population CDF and the corresponding CDF in Experiment 1 had the same shape (Kolmogorov-Smirnov, P = .0653 or P = .3876 after equalizing the individual medians), variance (Ansari-Bradley, P = .5975), and median (Kruskal-Wallis, P = .2664). In conclusion, this control confirmed fully the results of Experiment 1. Preventing the possibility to track the target with eye movements did not suppress the perceptual bias present when target tracking was possible.

**Table 7:** Median and JND values of the population CDFs for Experiment 6.

Parameter	Estimate	SE	Inferior CI	Superior CI
*Median*	10.2619129	0.2338829	9.8035109	10.720315
*JND*	0.8894454	0.1315613	0.6315899	1.147301

## General Discussion

**Figure 9:**
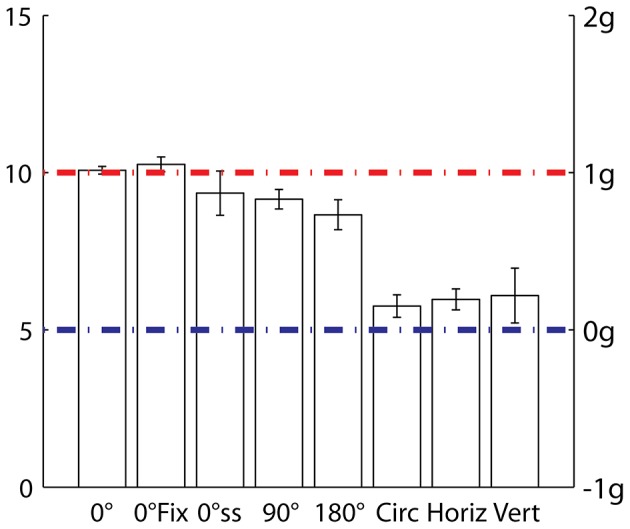
Means and standard errors (vertical bars) of the individual medians of the response histograms for all experiments. Motion conditions labeled as K_i_ and g-multiples in the left and right ordinates, respectively. Results from the different experiments are reported on the abscissae. 0°: 0° orientation, back-and-forth, Experiment 1. 0° Fix: 0° orientation, back-and-forth with eye fixation, Experiment 6. 0°ss: 0° orientation, curvilinear single-sweep, Experiment 2. 90°: 90° orientation, back-and-forth, Experiment 1. 180°: 180° orientation, back-and-forth, Experiment 1. Circ: circular periodic, Experiment 3. Horiz: linear horizontal unidirectional, Experiment 4. Vert: linear vertical unidirectional, Experiment 5.

The velocity profile perceived as most uniform depended on the target trajectory (Figure 9). In the experiments in which some features of the trajectory evoked the motion of either a simple pendulum or a mass-spring system (5 leftmost values in the bar-graph of Figure 9), observers tended to select kinematic profiles close to quasi-harmonic motion (K_10_, here denoted as 1g-kinematics), that is, the kind of motion naturally associated with these two physical systems. The bias towards quasi-harmonic motion was most consistent in the upright 0° orientation of Experiment 1, where both the gravitational pendulum and the rotational mass-spring system afford a plausible physical model with almost identical kinematics. When the predictions of the two dynamic models were mutually incompatible (90° and 180° orientation), the bias tended to diminish and to be less consistent across participants. Finally, in the experiments in which the trajectory was incompatible with both a simple pendulum and a rotational mass-spring system (3 rightmost values in Figure 9), the bias disappeared: observers tended to judge as the most uniform kinematic profile the one that was actually uniform. In the following, we consider the role of different mechanisms that can potentially account for these results.

### Eye movements

Eye movements can affect velocity judgments (see [47]). Thus, in the case of uniform motion, stimuli smoothly pursued by the eyes often appear slower than stimuli perceived while holding the gaze on a fixation point. Also, discrimination thresholds are higher in the former than the latter condition [48]. Moreover, natural kinematics (whether biological or inanimate) is easier to track with eye movements than kinematics deviating from natural models [42]–[43]. However, we can rule out that eye movement strategies played a major role in the perceptual bias towards quasi-harmonic motion because perceptual judgments were quite comparable in Experiment 1 (when participants were allowed to track target motion with eye movements) and in Experiment 6 (when they were asked to fixate; compare 0° and 0° Fix, respectively, in Fig. 9). The similarity of the results also rules out possible effects of presenting the target either in central (Experiment 1) or peripheral vision (Experiment 6).

### Role of adaptation

At the early stages of visual motion processing, velocity is estimated by comparing the outputs of a few spatio-temporal frequency channels whose sensitivities decay exponentially with a time constant of about 5–10 s [49]–[52]. Thus, a prolonged exposure to a moving pattern may affect the perceived velocity of subsequent stimuli [53]. In our experimental conditions we do not expect a net effect of long-term adaptation on perceptual judgments because the viewing time of each condition was significantly shorter. Potentially more relevant to our context is the observation [46] that perceived velocity monotonically decreases by about 5% within the first 500–750 ms of a uniform motion. Such a small motion adaptation may affect the perception of uniform motion in case of mildly accelerating stimuli, such as those in Experiments 3, 4 and 5 (Circ, Horiz and Vert in Figure 9). However, it can hardly account for the results with strongly accelerating stimuli in Experiments 1, 2 and 6, where target velocity changed by >150% over 420 ms in the 1g-condition (see Figure 1B). More generally, any form of adaptation should affect perception in the same manner in all experimental conditions involving the same target kinematics. Thus, low-level velocity processing cannot explain why stimuli with the same average velocity were perceived differently across conditions.

### Role of dynamic models

Experiment 1 extended to the case of curvilinear trajectories the anecdotal report by Johansson [4] that to-and-fro movements with velocity varying sinusoidally along a linear path look uniform. We found that 1g-kinematics was perceived as the most uniform motion at all 3 orientations (0°, 90° and 180°) of target trajectory. A parsimonious explanation of this result could be that the stimuli implicitly evoke the motion of a pendulum oscillating under a virtual gravity, displayed in arbitrary orientations relative to the observer. In other words, the 1g-kinematics would be the natural motion in a visual scene in which the oscillating pendulum and gravity are rotated coherently. Pendulum-like oscillations would provide a perceptual template acting as a baseline, so that velocity variations are reckoned only to the extent that they depart from this baseline. Although we cannot rule out this hypothesis, previous work [24], [53]–[54] showed that a mental rotation of gravity effects is possible in the presence of visual context cues, such as pictorial elements (e.g., trees, people) which are typically oriented relative to gravity, but it is much more difficult with scenes devoid of such cues [24], [27], [53] such as those used in our experiments.

A less parsimonious but more plausible explanation is that the stimuli of Experiment 1 evoked both physical models compatible with quasi-harmonic motion, namely a canonical pendulum under physical gravity and a mass-spring system. We argue that the relative strength of these two dynamic models could yield the subtle but significant differences in performance we found in Experiment 1 across orientations. When the target oscillated at 0° orientation, only the 1g-kinematics - among all the kinematic profiles we used - was consistent with the motion of a gravity pendulum (see Fig. 1). Indeed, whereas virtual gravity may be mentally rotated with the visual scene, physical gravity acts invariably in the downward direction. Moreover, although the rod of the pendulum was not shown, the displayed motion included several cues that could have evoked in the observers the motion of a gravity pendulum [22]. In fact, not only the quasi-harmonic velocity profile, but also the period and amplitude of the 1g-oscillations were consistent with those of the bob of a pendulum oscillating along a circular arc with the appropriate radius (Figure 2A). At the same time, however, the 1g-kinematics was also consistent with the motion of a mass interacting with a rotational spring when gravity is irrelevant. Actually, in the small angle approximation, the effects expected from the action of physical gravity and of an elastic restoring force are indistinguishable. Thus, the two expectations reinforced mutually and resulted in the strongest and most consistent bias towards the quasi-harmonic motion.

Instead, when the target oscillated at 90° or 180° orientation, the 1g-kinematics (accelerating toward the trajectory midpoint and decelerating away from it) was still compatible with a mass-spring system, but became incompatible with the action of physical gravity. In fact, for trajectories at 180° orientation, gravity would decelerate the target toward the trajectory midpoint and accelerate it away from the midpoint, whereas at 90° orientation gravity would accelerate the target on the way down and decelerate it on the way up. We suggest that, in these conditions, the mass-spring model is the dominant one and is a sufficiently strong prior to attract the perceptual responses much closer to 1g-kinematics than to 0g-kinematics even at these orientations. However, we also argue that the powerful pendulum model is still somehow evoked. The shift of the perceptual bias slightly away from the 1g-kinematics and towards the 0g-kinematics, and the larger variability across trials and participants with respect to the 0° orientation (see Figure 2) is then interpreted as the consequence of the conflict between these two incompatible models. Note that our interpretation is not critically dependent on whether the dynamic model evoked by the stimuli is inanimate, such as a swinging pendulum or branch of a tree, or it is animate, such as a human arm or leg swinging back-and-forth during walking, because the typical driving forces are gravitational and elastic in both cases. Although we have emphasized the role of a dynamic prior (i.e., related to the forces underlying motion), it could be argued that the relevant prior is kinematic [15]. In other words the visual system might be tuned to simple harmonic motion *per se*. This however would not provide a simple account for the significant differences emerging among the different orientations in Experiment 1.

### Role of target trajectory in global motion integration

The comparison of the results of Experiment 1 and Experiment 2 (0° and 0°ss Figure 9) shows that the quasi-harmonic velocity modulation of the 1g-condition continues to be perceived as constant (although with a larger scatter of the responses) even when inversions of movement direction are no longer present (Experiment 2) but the curvilinear shape of the trajectory still evokes a pendulum motion. Instead, the simple change of the path from curvilinear (Experiment 2) to rectilinear unidirectional, either horizontal (Experiment 4) or vertical (Experiment 5), changed drastically the perceptual judgments. In these two experiments, we found that, although on average observers chose a slightly accelerated motion condition (K_6_), they were often able to correctly identify K_5_ as the constant velocity profile (Figure 6 and 7). Apparently, when the motion is unidirectional, a curved trajectory, along with the implied pivot, is a mandatory feature of the stimulus in order to evoke an oscillating model. As in Runeson's experiment [3], unidirectional linear movements may instead evoke the motion of a mass following an initial force pulse. Our stimuli accelerated towards the trajectory midpoint and decelerated thereafter. Instead the stimuli employed by Runeson accelerated at the onset of the sweep and then leveled off to a constant velocity. In spite of this difference, the small bias towards a slightly accelerated motion with rectilinear unidirectional motion (Experiment 4 and 5) is consistent with Runeson's statement that, to be seen as constant, a uni-directional motion must be mildly accelerated at the onset [3].

Albeit necessary, curved trajectories are not sufficient to evoke an oscillatory model. In Experiment 3 the target moved along a circle without ever changing direction, and was visible only in the bottom and top quadrants. With this stimulus most observers chose a slightly accelerated motion condition (K_6_), but they were often able to correctly identify K_5_ as the constant velocity profile (Figure 5B). A factor that may play a role in this case is that the two segments of trajectory shown sequentially could be perceived as a partially occluded circular motion, because the timing was compatible with this interpretation. The simplest physical system compatible with such a trajectory would be a mass moving under the action of a central force. This could correspond, for instance, to the visual experience of a stone tied to a string being rotated in air. For such a system, angular velocity is constant, consistent with the perceptual responses we found in several cases.

### Event perception

We believe that the effects of target trajectory on the perception of uniform velocity are best explained in the context of event perception [55]–[57]. According to Gibson [13], the perception of dynamic events is afforded by the spatiotemporal visual patterns that are invariantly and characteristically associated to the events. These patterns need not include many morphological details. Indeed, several categories of events, such as moving animate organisms [58] or trees shaken by the wind [59], are easily identified based on point-light displays. Even abstract events represented by geometrical forms are discriminated on the basis of the spatio-temporal trajectory of a single moving dot [60]–[61].

In previous studies, event recognition appeared often disrupted by inversion of the trajectory relative to the direction of gravity, as in the case of detection of biological motion [62] or inanimate pendulum motion [24]. Here, instead, the perceptual performance with the upside-down trajectory (180° orientation in Experiment 1) was more variable, but did not differ drastically relative to that with the upright trajectory (see Fig. 2). However, our observers were not asked explicitly to identify the target motion with a pendulum or any other specific physical system, but only to indicate the kinematic profile which looked more uniform. We argue that the perception of uniform motion is linked with the implicit detection of a specific natural event underlying the observed kinematics, but does not necessarily require the explicit recognition of the event.

### Summary and conclusions

Taken together, the results of all our experiments are compatible with the hypothesis put forth by Runeson [3] that the perception of dynamic stimuli is biased by the laws of motion obeyed by natural events, so that only natural motions appear uniform. Our data show further that target trajectory is a critical factor in eliciting expectations about the underlying dynamics. The implied dynamic model switched from a simple gravity pendulum to a mass-spring system to a rotating mass depending on the global path traced by the target. Subtle features of the spatio-temporal trajectory of a single moving dot are sufficient to elicit an expectation about the underlying dynamics of a plausible physical event [60]–[61].

According to current views on motion perception, velocity might be estimated in the brain by means of a process equivalent to a Bayesian combination of on-line sensory measurements (providing the likelihood of the estimate) with a prior related to the statistical distribution of velocities in the natural world [63]. Both the sensory evidence and the prior are weighed in inverse proportion to the variance of the corresponding signal. In the context of the present experiments, the prior would be related to the natural dynamics implied by the target trajectory. If so, the tendency observed in different conditions to perceive as uniform the quasi-harmonic motion of the 1g-condition (despite velocity changes greater than 150%) should be interpreted as resulting from a strong (low variance) prior of pendulum or mass-spring dynamics.

## Supporting Information

File S1 **Contains: Movies S1–S21.** Down-sampled (50 Hz) version of the stimuli presented in the 0° orientation condition of Experiment 1, (see schematic of Figure 2A). The movies correspond to the 21 velocity profiles referred to in the text as [K_0_, K_1_,....K_20_]. Movie S1 corresponds to K_0_ (−1 g), Movie S6 to K_5_ (0 g), Movie S11 to K_10_ (1 g), Movie S16 to K_15_ (2 g), and Movie S21 to K_20_ (3 g). Because each movie shows only one stimulus period, to see the stimuli repeatedly the media player must be set in reply (continuous) mode.
(ZIP)

File S2 **Contains: Movies S22–S42.** Stimuli presented in the 180° orientation condition of Experiment 1 (Figure 2A). Movie S22 corresponds to K_0_ (−1 g), and Movie S42 to K_20_ (3 g).
(ZIP)

File S3 **Contains: Movies S43–S63.** Stimuli presented in Experiment 4. Movie S43 corresponds to K_0_ (−1 g), and Movie S63 to K_20_ (3 g).
(ZIP)
